# Association Between Web-Based Physician Ratings and Physician Disciplinary Convictions: Retrospective Observational Study

**DOI:** 10.2196/16708

**Published:** 2020-05-14

**Authors:** Jessica Janine Liu, Hanna R Goldberg, Eric JM Lentz, John Justin Matelski, Asim Alam, Chaim M Bell

**Affiliations:** 1 Division of General Internal Medicine Department of Medicine University of Toronto Toronto, ON Canada; 2 Department of Medicine University Health Network Toronto, ON Canada; 3 Department of Obstetrics and Gynecology University of Toronto Toronto, ON Canada; 4 Faculty of Medicine McMaster University Hamilton, ON Canada; 5 Department of Anesthesia and Surgery North York General Hospital Toronto, ON Canada; 6 Department of Laboratory Medicine and Pathobiology University of Toronto Toronto, ON Canada; 7 Sinai Health System Toronto, ON Canada

**Keywords:** quality improvement, patient satisfaction, patient-centered care

## Abstract

**Background:**

Physician rating websites are commonly used by the public, yet the relationship between web-based physician ratings and health care quality is not well understood.

**Objective:**

The objective of our study was to use physician disciplinary convictions as an extreme marker for poor physician quality and to investigate whether disciplined physicians have lower ratings than nondisciplined matched controls.

**Methods:**

This was a retrospective national observational study of all disciplined physicians in Canada (751 physicians, 2000 to 2013). We searched ratings (2005-2015) from the country’s leading online physician rating website for this group, and for 751 matched controls according to gender, specialty, practice years, and location. We compared overall ratings (out of a score of 5) as well as mean ratings by the type of misconduct. We also compared ratings for each type of misconduct and punishment.

**Results:**

There were 62.7% (471/751) of convicted and disciplined physicians (cases) with web-based ratings and 64.6% (485/751) of nondisciplined physicians (controls) with ratings. Of 312 matched case-control pairs, disciplined physicians were rated lower than controls overall (3.62 vs 4.00; *P*<.001). Disciplined physicians had lower ratings for all types of misconduct and punishment—except for physicians disciplined for sexual offenses (n=90 pairs; 3.83 vs 3.86; *P*=.81). Sexual misconduct was the only category in which mean ratings for physicians were higher than those for other disciplined physicians (3.63 vs 3.35; *P*=.003)

**Conclusions:**

Physicians convicted for disciplinary misconduct generally had lower web-based ratings. Physicians convicted of sexual misconduct did not have lower ratings and were rated higher than other disciplined physicians. These findings may have future implications for the identification of physicians providing poor-quality care.

## Introduction

### Background

The ability of patients to accurately evaluate health care quality is not well understood. Although some studies demonstrate an association between greater patient satisfaction and quality of care, others show either no relationship or even poorer outcomes with increased patient satisfaction [1-12]. Over the last decade, with the advent of physician rating websites such as healthgrades.com, ratemds.com, and vitals.com, a novel source of patient satisfaction data has emerged. Such websites have become popular forums for patients to evaluate and publicly share their health care experience.

Previous studies have focused on the awareness of and frequency of ratings for specific medical specialties on rating websites in Canada, the United States, China, and Germany [11-29]. The recent focus has been to correlate web-based ratings with quality outcomes or surrogates such as postoperative mortality—with variable findings [13,30-35]. Web-based physician ratings represent a novel, unsolicited data source of the patient experience with health care providers that is unique from more traditional satisfaction measures, such as solicited surveys.

Physician misconduct can be considered a reflection of poor quality care. Physicians are investigated, convicted, and disciplined by their professional associations for activities such as unprofessional behavior, sexual misconduct, failure to meet standards of care, fraud, abuse of drugs and alcohol, and negligence. Resultant penalties range from fines and mandatory education to license suspension and revocation. Although disciplinary proceedings are publicly posted by each province’s physician regulatory college, at the time of a clinical encounter, patients are often unaware of a physician’s disciplinary history.

### Objectives

We were interested in whether or not physicians who have been convicted and punished for misconduct are rated differently than nondisciplined physician controls. We hypothesized that, for many types of misconduct, patients would accurately recognize poor-quality physicians and felt that, overall, disciplined physicians would have lower web-based ratings than controls. We also sought to determine whether ratings were consistently lower across all types of misconduct, and we hypothesized that associations between ratings and discipline would differ depending on the type of misconduct.

## Methods

### Physician Databases: Disciplined Physicians

This retrospective cohort study reviewed publicly available information on physician disciplinary proceedings published by Canadian provincial and territorial physician regulatory colleges. A database from January 2000 to December 2013 was compiled (described previously) [36-40]. We collected demographic information for every disciplined physician in the country, including gender, license type (independent vs educational), medical school (ie, North American trained versus international medical graduate [IMG]), year of graduation, and specialty. We collected information on types of misconduct and resultant penalties that were determined by the provincial colleges. Misconduct was categorized into (1) inappropriate prescribing, (2) criminal conviction, (3) fraudulent behavior or prevarication, (4) misconduct secondary to mental illness, (5) self-use of drugs or alcohol, (6) sexual misconduct, (7) practice below standard of care, (8) unprofessional conduct, (9) unlicensed activity, (10) miscellaneous findings (ie, improper maintenance of medical records and confidentiality breaches), and (11) unclear. Punishments included (1) license revocation, (2) voluntary license surrender, (3) suspension, (4) license restriction, (5) mandated retraining, education or assessment, (6) mandated participation in psychological counseling or addiction rehabilitation, (7) formal reprimand, (8) fine or cost repayment, and (9) other [36-42].

### Cases and Controls

We employed a nested case-control design and matched each disciplined physician (*cases*) with a nondisciplined counterpart (*controls*) according to specialty, gender, town of listed practice, and years in medical practice (within 5 years). We developed a group of nondisciplined physician controls by searching provincial physician regulatory college websites for each disciplined physician and narrowing our search terms by the abovementioned criteria. In certain instances (ie, 2 provinces), if after controlling for the 4 matching criteria, multiple physician matches were possible, a physician was chosen at random. In total, 751 disciplined physicians were matched with 751 nondisciplined controls. A nondisciplined control was found for every disciplined physician (Table 1).

**Table 1:** Characteristics of cases (751 disciplined physicians; 2000-2013) and controls (751 nondisciplined physicians, matched for gender, years since graduation, and city of practice [where possible]).

Physician characteristic		Cases (disciplined physicians; N=751)			Controls (nondisciplined controls; N=751)			
		Rated matched (n=312)	Rated unmatched (n=159)	Unrated (n=280)		Rated matched (n=312)	Rated unmatched (n=173)	Unrated (n=266)
**Sex, n (%)**								
	Female	28 (9.0)	14 (8.8)	24 (8.6)		28 (9)	15 (8.7)	23 (9.6)
	Male	284 (91)	145 (91.2)	256 (91.4)		284 (91)	158 (91.3)	243 (91.4)
Years in practice since graduation, mean (SD)		27.9 (10.4)	29.9 (11.7)	30.7 (12.0)		27.3 (10.9)	27.2 (11.4)	29.8 (11.9)
**Specialty, n (%)**								
	Family medicine	180 (57.7)	94 (59.1)	167 (59.6)		180 (57.7)	113 (65.3)	152 (57.1)
	Internal medicine	7 (2.2)	2 (1.3)	14 (5.0)		6 (1.9)	8 (4.6)	10 (3.8)
	Obstetrics	24 (7.7)	2 (1.9)	5 (1.8)		24 (7.7)	5 (2.9)	3 (1.1)
	Pediatrics	6 (1.9)	5 (3.1)	2 (0.7)		6 (1.9)	1 (0.6)	6 (2.3)
	Psychiatry	23 (7.4)	23 (14.5)	46 (16.4)		23 (7.4)	19 (11.0)	47 (17.7)
	Radiology	0 (0)	1 (0.6)	3 (1.1)		1 (0.3)	2 (1.2)	2 (0.8)
	Surgery	23 (7.4)	7 (4.4)	25 (8.9)		24 (7.7)	20 (11.6)	13 (4.9)
	Other	49 (15.7)	20 (12.5)	12 (4.3)		48 (15.4)	4 (2.3)	22 (8.2)
**Medical school, n (%)**								
	International medical graduate^a^	91 (29.2)	50 (31.4)	108 (38.6)		104 (33.3)	59 (34.1)	113 (42.4)
	North American graduate	221 (70.8)	109 (68.6)	172 (61.4)		208(66.7)	14 (65.9)	153 (57.5)
Number of ratings, mean (SD)		19.6 (13.0)	15.0 (11.82)	N/A^b^		15.5 (11.5)	14.9 (10.5)	N/A
Overall rating, mean (SD)		3.62 (0.82)	3.42 (0.98)	N/A		4.00 (0.75)	3.91 (0.82)	N/A

^a^International medical graduate denotes physicians who graduated from a non-Canadian or non-US medical school.

^b^N/A: not applicable.

### Physician Ratings Data

RateMDs.com is a publicly accessible physician rating website founded in the United States in 2004. Since its launch in Canada (2005), it is the country’s leading physician rating website and one of the most popular physician rating websites in North America [41,43]. As of 2013, RateMDs.com included more than 640,000 ratings of over 57,000 unique physicians in Canada [29]. No registration or subscription is required to view or submit a rating, and there are no monetary reimbursement or other incentives to rate a physician. Physicians are rated on staff (typically front office staff), punctuality, helpfulness, and knowledge, on a scale of 1 to 5 (1=*terrible*, 2=*poor*, 3=*okay*, 4=*good*, and 5=*excellent*). Raters may provide text comments if desired. It must be noted that RateMDs.com does not provide disciplinary information. We reviewed all disciplined and nondisciplined control physicians on this website and recorded rating scores. Data collection took place between approximately May 2014 and September 2014, with data cleaning and quality control performed by a second party in July 2015.

### Creation of the Dataset

**Figure 1:**
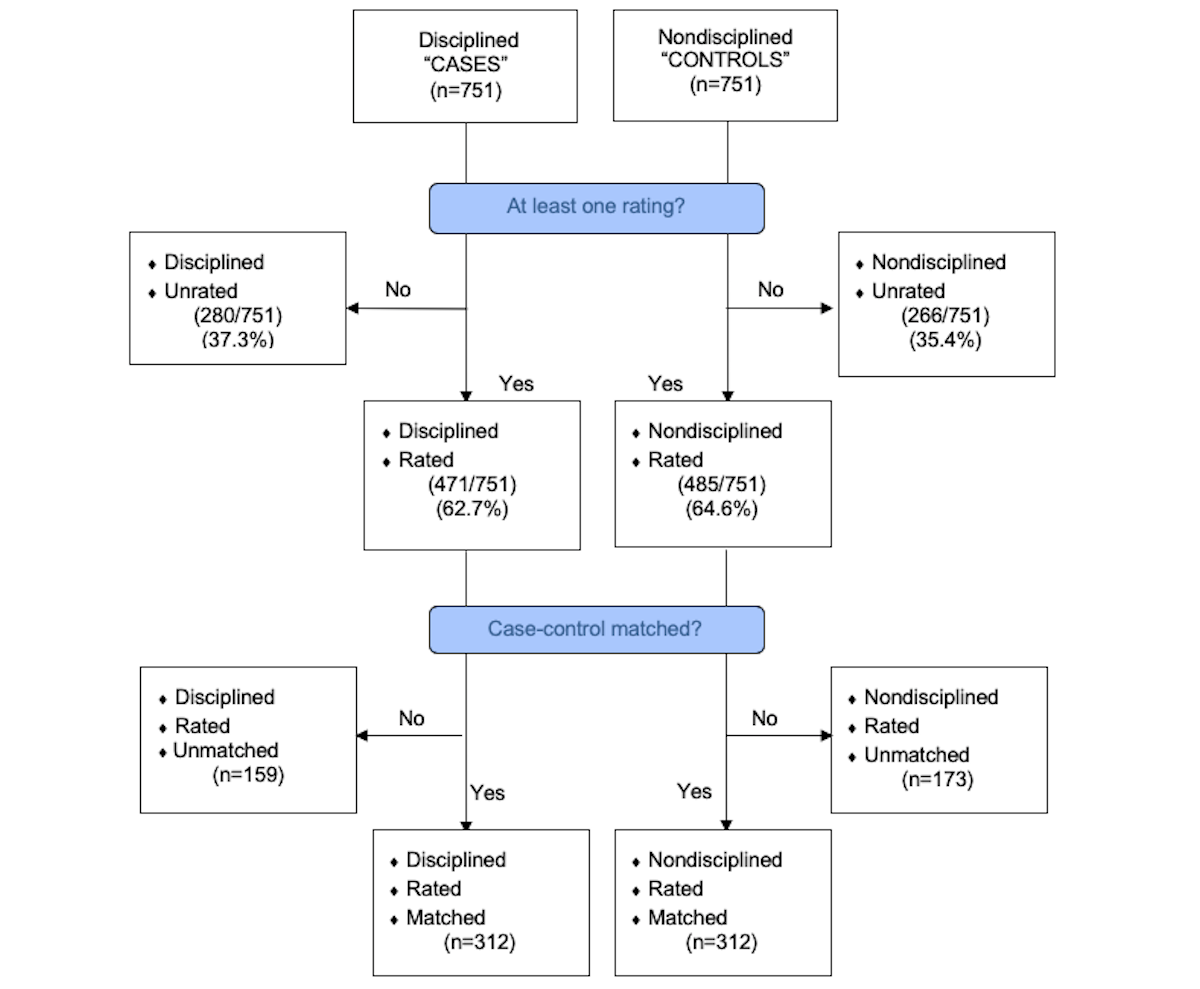
Flow chart for developing matched case (disciplined) and control (nondisciplined) groups of physicians, according to the presence of at least one web-based rating.

We paired 751 disciplined physicians with 751 nondisciplined matched controls and collected information from rateMDs.com for each physician. As not all physicians were rated, this resulted in 4 groups: disciplined rated *cases*, disciplined unrated, control rated, and control unrated. When considering *pairs* of cases and controls who both had web-based ratings, our dataset included 312 physician pairs (Figure 1). We used only matched pairs for analysis and performed analyses when there were more than 50 case-control matched pairs. We also grouped disciplined physicians according to types of misconduct and punishments. The number of matched pairs available for testing varied from 2 to 254 pairs available for comparison (Table 2).

**Table 2:** Mean web-based physician ratings of disciplined physicians (2000-2013) and nondisciplined controls (2000-2013) by the type of misconduct and punishment.

Description		Case-control pairs	Disciplined physicians (cases)			Nondisciplined matched controls			Statistical analysis
			Mean rating	95% CI			Mean rating	95% CI	Wald *P* value
Overall		312	3.62	3.53-3.71			4.00	3.91-4.08	<.001
Physicians with more than one conviction (repeat offenders)		44	3.44	3.21-3.85			4.00	3.67-4.33	<.01
**Misconduct**									
	Standard of care breach	113	3.57		3.42-3.72		3.98	3.98-3.84	<.001
	Inappropriate prescribing	55	3.50		3.29-3.71		3.97	3.79-4.16	.001
	Unlicensed activities	35	3.42		3.18-3.66		4.35	4.16-4.54	N/A^a^
	Sexual misconduct	90	3.83		3.67-3.99		3.86	3.68-4.04	.81
	Mental illness	2	3.89		3.61-4.17		3.26	3.05-3.47	N/A
	Drugs/alcohol abuse	8	4.25		3.94-4.55		3.74	3.29-4.20	N/A
	Fraudulent behavior	39	3.44		3.18-3.70		4.11	3.89-4.32	N/A
	Conviction of a crime	13	3.59		3.13-4.04		4.27	3.95-4.59	N/A
	Unprofessional behavior	62	3.43		3.22-3.64		4.03	3.86-4.21	<.001
	Miscellaneous	79	3.45		3.29-3.89		3.99	3.82-4.17	<.001
**Punishment**									
	License revocation	25	3.59		3.29-3.89		4.21	3.91-4.51	N/A
	License surrender	8	3.98		3.41-4.55		3.38	2.67-4.09	N/A
	License suspension	191	3.71		3.60-3.83		3.99	3.88-4.10	<.001
	Restriction	115	3.69		3.55-3.83		3.91	3.78-4.05	.03
	Mandatory retraining	120	3.61		3.48-3.73		3.98	3.85-4.12	<.001
	Counseling	43	3.58		3.35-3.80		3.78	3.42-4.03	N/A
	Formal reprimand	144	3.58		3.45-3.71		4.02	3.89-4.14	<.001
	Other punishment	34	3.52		3.26-3.78		4.04	3.85-4.22	N/A
	Fine	254	3.60		3.50-3.70		4.02	3.93-4.11	<.001

^a^N/A: not applicable (when there were less than 50 case-control pairs available for comparison, analysis was not completed).

### Statistical Analysis

#### Analysis 1: Comparison of Disciplined Versus Nondisciplined Physicians (Matched Analysis)

To compare ratings between disciplined and nondisciplined physicians, we computed an overall average rating for each physician using the mean of the available rating categories, then calculating an overall weighted mean. Generalized estimating equations (GEEs) were used to estimate the average rating by group (disciplined vs nondisciplined), and GEES were used for each type of misconduct or penalty. GEEs were selected to account for the matched study design. We felt it was appropriate to select GEEs over nonparametric testing, given that there were sufficiently large (eg, 451) distinct average ratings, and therefore, it could treat ordinal data similar to continuous data. This analysis allowed us to report 95% CIs for the estimated group means and provide a sense of the precision of the estimates in addition to significance testing. We reported the estimated mean ratings by group, 95% CIs for these estimates, and Wald *P* value against the null hypothesis (no group difference). An α of .05 was used as the threshold for statistical significance. Analyses were performed using the *geepack* package in R version 3.0.3 (R Foundation).

#### Analysis 2: Comparison of Physicians Disciplined for a Specific Type of Misconduct/Punishment Versus the Rest of the Disciplined Physicians Cohort

Recognizing that the severity of physician misconduct and punishment is variable (eg, ranging from substandard recordkeeping to more egregious offenses such as sexual misconduct), we compared physician ratings for specific disciplinary offenses with those of the *at large* disciplined physicians cohort. Mixed effects models were used for analyses of ratings among disciplined physicians, considering each physician’s overall average web-based rating and category-specific ratings as outcomes. The presence of each type of misconduct/punishment in a physician’s discipline record was used as a binary predictor. Gender, year of offense, province, professional years, and IMG status were included as fixed effects, and physician specialty was included as a random effect. The estimates reflect the mean centering of the year of offense and professional relative to the rest of the disciplined cohort. We report the estimated mean ratings by group, 95% CIs for these estimates, and Wald *P* value against the null hypothesis (no group difference). An α of .05 is used as the threshold for statistical significance. Analyses were performed using the *nlme* package in R version 3.0.3.

### Sensitivity Analysis

To assess the degree to which physicians with a low overall number of ratings (ie, <5 or <10 ratings) influenced our overall results, we performed additional testing on both analyses 1 and 2 by excluding instances in which physicians had (1) less than 5 overall ratings and (b) less than 10 overall ratings.

## Results

### Disciplined Physicians Versus Nondisciplined Physicians: Matched Analysis

We paired 751 disciplined physicians with 751 nondisciplined matched controls. Of the 751 disciplined physicians, 37.3% (280/751) did not have any web-based ratings, whereas 62.7% (471/751) had at least one rating. Of the 751 nondisciplined physician controls, 64.6% (485/751) had at least one rating, whereas 35.4% (266/751) were not rated online. When comparing rated, but unmatched, physicians, 21.1% (159/751) were disciplined, rated, but unmatched compared with 23.0% (173/751) nondisciplined, rated, but unmatched. When considering *pairs* of cases and controls who both had ratings, our dataset included 312 physician pairs (Figure 1). When we grouped disciplined physicians according to the types of misconduct and punishments, the number of matched pairs available varied, ranging from 2 to 254 available pairs (Table 2).

When we compared the 312 pairs of convicted and disciplined physicians with nondisciplined controls, disciplined physicians were rated lower than nondisciplined physicians for all offenses and punishments (mean rating 3.62, SD 0.82 vs mean rating 4.00, SD 0.75; *P*<.001). When comparing rated, but unmatched, physicians, disciplined unmatched physicians had even lower ratings than nondisciplined unmatched physicians (mean 3.42, SD 0.98 vs mean 3.91, SD 0.82). As 12.5% (94/751) of our disciplined physicians cohort had more than one disciplinary conviction during our study period, we also looked at this group of *repeat offenders*. Of the 94 disciplined physicians who were repeat offenders, approximately half were available for case-control analysis, as 44 disciplined physicians were appropriately matched to a case-control where both groups had ratings. Disciplined repeat offenders had mean ratings that were also lower than controls (mean 3.44, SD 4.09 vs mean 4.00, SD 0.81; *P*<.01).

The mean rating for disciplined physicians was lower than that for nondisciplined physician–matched controls for the following types of misconduct and punishment: standard of care breach (113 pairs; 3.57 vs 3.98; *P*<.001), inappropriate prescribing (55 pairs; 3.50 vs 3.97; *P*<.001), unprofessional behavior (62 pairs; 3.43 vs 4.03; *P*<.001), miscellaneous/unclear (79 pairs; 3.45 vs 3.99; *P*<.001), license suspension (191 pairs; 3.71 vs 3.99; *P*<.001), license restriction (115 pairs; 3.69 vs 3.91; *P*=.027), mandatory retraining (120 pairs; 3.61 vs 3.98; *P*<.001), formal reprimand (144 pairs; 3.58 vs 4.02; *P*<.001), and fine (254 pairs; 3.60 vs 4.02; *P*<.001; Table 1). No significant differences were detected for physicians who were disciplined for sexual offenses, compared with nondisciplined matched controls (n=90 physician pairs; 3.83 vs 3.86; *P*=.81; Table 2).

### Comparison of Specific Type of Misconduct/Punishment Versus the Rest of the Disciplined Physicians Cohort

Sexual misconduct was the only category of misconduct in which mean ratings for this group of physicians were higher than those for other disciplined physicians. Moreover, 62.7% (471/751) disciplined physicians who were rated online, 219 were disciplined for sexual misconduct. The overall mean rating of physicians disciplined for sexual misconduct was higher than that of all other disciplined physicians (3.63; 95% CI 3.17-4.08 vs 3.35, 95% CI 2.91-3.80; *P*=.003). This overall effect was consistent and significant across all 4 rating subcategories (*staff*: 3.93 vs 3.71; *P*=.023; *punctuality*: 3.60 vs 3.36; *P*=.011; *helpfulness*: 3.83 vs 3.47; *P*<.001; and *knowledge*: 4.02 vs 3.66; *P*<.001; Multimedia Appendix 1). Physicians disciplined for fraudulent behavior and miscellaneous had *lower* overall ratings when compared with other disciplined physicians (fraudulent behavior: 3.15 vs 3.44; *P*=.01 and miscellaneous: 3.31 vs 2.51; *P*=.04). For punishments, suspension was the only type of punishment in which this group of disciplined physicians was rated higher than all other disciplined physicians (3.54 vs 3.33; *P*=.023); however, this result did not remain robust when physicians with less than 10 ratings were excluded from our sensitivity analysis. For all other types of misconduct and punishments, no overall mean rating differences existed compared with all other disciplined physicians (Multimedia Appendix 1).

### Sensitivity Analysis

Of 312 cases and 312 controls, 48 case physicians and 68 control physicians had less than 5 ratings. Similarly, 84 case physicians and 117 control physicians had less than 10 ratings. To assess whether such ratings influenced our main results, we performed sensitivity analyses by excluding cases in which physicians had (1) less than 5 and (2) less than 10 ratings. Our main results remained robust (Multimedia Appendices 2 and 3). When we excluded physicians with few ratings, our finding that disciplined physicians had lower overall mean ratings did not change (<5 ratings: 3.61 vs 4.01; *P*<.001 and <10 ratings: 3.52 vs 4.01; *P*<.001). When broken down by type of misconduct and punishment, results also remained robust—that is, disciplined physicians had lower ratings than nondisciplined case-controls, with the exception of sexual misconduct (<5 ratings and sexual misconduct: 3.89 vs 3.94; *P*=.69 and <10 ratings and sexual misconduct: 3.79 vs 3.92; *P*=0.36).

Similarly, when comparing physicians disciplined for types of misconduct with all other disciplined physicians, all results remained robust, with 2 minor exceptions. Ratings for physicians whose licenses were suspended no longer differed from all other disciplined physicians, nor did the ratings for physicians who were punished with a formal reprimand (Multimedia Appendix 3). All other results remained consistent after sensitivity analyses.

## Discussion

### Principal Findings

Our study used a national dataset of all disciplined physicians and collected their available online ratings from rateMDs.com over a 10-year period. Of over 750 matched physician pairs, 63.6% (956/1502) physicians are rated online. For most types of misconduct, disciplined physicians are rated lower than nondisciplined controls. However, physicians disciplined for sexual misconduct were not rated differently than controls and, in fact, were rated *higher* when compared with all other disciplined physicians, a directional relationship that was not found with any other type of misconduct.

### Comparison With Prior Work

Our results are in general agreement with other studies that show that physicians are, overall, rated positively [13,20,21,29]. Our findings are also consistent with data showing lower online ratings for physicians on probation for many types of misconduct, but not sexual offenses [34]. There may be something unique about physicians who commit sexual misconduct that distinguishes them from other convicted physicians, at least with respect to online ratings.

We found that online raters discerned a difference between disciplined and nondisciplined physicians with respect to online ratings overall; however, interestingly, sexual misconduct was the only category in which this effect was not seen. Furthermore, we found that physicians who were disciplined for sexual misconduct are rated *more favorably* than the rest of the disciplined physician cohort. Again, sexual misconduct was the only category of misconduct in which mean ratings were higher than all other disciplined physicians.

Our findings related to sexual offense convictions are consistent with previous findings. Only a handful of studies have compared sexual offender physicians with other physicians; however, it has been reported that some antisocial personality traits were unique to psychiatrists who were subsequently convicted of sexual boundary violations and that these characteristics were identifiable early in training [44-47].

This study adds to the body of literature on online physician ratings and extends current knowledge to include extremes of poor quality (ie, physician disciplinary convictions). This is the first study to combine 2 large, comprehensive national databases of physician discipline and web-based physician ratings over a 13-year period, using a rigorous matched control approach. We highlight the heterogeneity of disciplined physicians as a group and are among the first to identify this finding in physician sexual offenders. Although the majority of low-rated physicians are not disciplined and they are not sexual offenders, we feel that the potential for patient harm is sufficient enough in such cases to warrant further investigation of this group of disciplined physicians. Future studies could focus on predicting or developing interventions to prevent patient harm.

### Limitations

We recognize several limitations. First, our study assumes that disciplined physicians, as a group, are poor-quality physicians. Although not perfectly synonymous, these physicians have been convicted by their professional colleges for conduct that is substandard, inappropriate, or morally not in line with professional standards. As such, this is an excellent surrogate for poor quality. Second, we cannot exclude that publicly posted ratings may, themselves, influence future ratings. Although we considered censoring ratings after a particular disciplinary proceeding became a public record, we felt a time-based analysis would decrease the number of ratings in our analysis, with no clear added benefit against potential bias. Moreover, the uncertainty of whether the rater had advance knowledge of the physician would remain, as it would be difficult to ascertain whether raters were influenced by other sources (eg, popular media attention). Interestingly, we found that physicians who were disciplined for sexual misconduct (the misconduct category frequently reported in the media) were rated no differently than controls. In fact, they were rated *higher* when compared with the rest of disciplined physicians, making us more likely to accept our findings. Although occasionally there was a mention of misconduct in the comments, we estimated this to reflect a small proportion (ie, <5%) of all comments. Moreover, we would argue that for our research question, timing may be less relevant, that is, a physician disciplined in 2000 and reviewed in 2005 versus a physician who was reviewed in 2000 and disciplined in 2005 are both relevant enough to merit consideration.

Third, although we used a stringent matching process, in smaller centers, it was not possible to match by subspecialty for 5 physicians. In this case, we matched as closely as possible (ie, we matched surgeons with another surgeon rather than, eg, a psychiatrist). This represented less than 1% of cases. Fourth, as not all physicians are rated on websites, data may not be generalizable. However, 63.6% (956/1502) of physicians had an online presence, which is much higher than in previous studies [13,15,31], and when we analyzed data from disciplined, unmatched physicians, overall demographics and mean ratings did not substantially differ. In fact, ratings of unmatched, disciplined physicians were lower than unmatched, undisciplined physicians. We also considered external validity concerns in potential comparisons between the 60% of physicians who are rated online versus those who are unrated. However, because our physician control group was hand selected to resemble the disciplined physician group, and not representative of the general population, such comparisons would not be particularly useful; therefore, we specifically refrained from making such direct comparisons between rated and unrated physicians. Finally, rating website users may be different with respect to access to a computer and inclination to post online ratings. However, this is an issue germane to all online ratings. Taken together, we feel that these limitations would not significantly alter our conclusions.

### Conclusions

Disciplined physicians are rated lower than control physicians by those who rate their physicians online, in keeping with the hypothesis that patients can accurately appraise health care quality. However, any ability to ascertain quality becomes more difficult for physicians disciplined for sexual misconduct. Our findings suggest that this group of physicians deserves further investigation to better understand why they would be rated more favorably than all other disciplined physicians. Our research may have implications for the identification of at-risk physicians to develop interventions before patient harm can occur [48].

## Appendix

Multimedia Appendix 1 Physicians disciplined for specific episodes of misconduct, as compared with other disciplined physicians (2000-20013), broken down by website rating category.

Multimedia Appendix 2 Sensitivity Analysis. Mean online ratings (with 95% confidence intervals) for cases versus controls, using inclusion cut-offs of at least 1 online rating (original analysis), at least 5 ratings or at least 10 ratings per physician.

Multimedia Appendix 3 Sensitivity Analysis. Mean online ratings for physicians disciplined for specific types of misconduct, as compared with all other disciplined physicians, using inclusion cut-offs of at least 1 online rating, at least 5 ratings or at least 10 ratings per physician.

## Abbreviations

GEEs: generalized estimating equations
IMG: international medical graduate
