# Supplementary material for: Association Between Web-Based Physician Ratings and Physician Disciplinary Convictions: Retrospective Observational Study
Source: J Med Internet Res. 2020 May 14;22(5):e16708. doi: 10.2196/16708 (PMC7256745; doi:10.2196/16708)
Supplement: Multimedia Appendix 1 [file jmir_v22i5e16708_app1.docx]

| **Category of**  **misconduct** | **Website rating domain** | **Physicians disciplined for specific misconduct** | **Other disciplined physicians** | **Test statistic (*P*-value)** |
| --- | --- | --- | --- | --- |
| Standard of care breach | Number of physicians | 243 | 508 |  |
|  | Staff | 3.72 (3.24-4.19) | 3.64 (3.18-4.11) | 0.47 |
|  | Punctuality | 3.17 (2.71-3.63) | 3.12 (2.67-3.56) | 0.53 |
|  | Helpfulness | 3.39 (2.86-3.92) | 3.42 (2.90-3.94) | 0.78 |
|  | Knowledge | 3.56 (3.04-4.08) | 3.61 (3.10-4.12) | 0.65 |
|  | **OVERALL MEAN** | **3.46 (3.00-3.92)** | **3.45 (3.00-3.90)** | **0.92** |
|  |  |  |  |  |
| Inappropriate prescribing | Number of physicians | 114 | 637 |  |
|  | Staff | 3.58 (3.07-4.08) | 3.67(3.21-4.12) | 0.43 |
|  | Punctuality | 3.09 (2.60-3.57) | 3.13 (2.69-3.58) | 0.66 |
|  | Helpfulness | 3.50 (2.94-4.07) | 3.41 (2.90-3.92) | 0.47 |
|  | Knowledge | 3.63 (3.07-4.18) | 3.60 (3.09-4.10) | 0.77 |
|  | **OVERALL MEAN** | **3.45 (2.96-3.94)** | **3.45 (3.01-3.90)** | **0.98** |
|  |  |  |  |  |
| Unlicensed activity | Number of physicians | 79 | 672 |  |
|  | Staff | 3.56 (3.04-4.08) | 3.67 (3.21-4.13) | 0.41 |
|  | Punctuality | 3.11 (2.60-3.61) | 3.13 (2.69-3.58) | 0.83 |
|  | Helpfulness | 3.21 (2.63-3.79) | 3.42 (2.91-3.94) | 0.16 |
|  | Knowledge | 3.37 (3.80-3.94) | 3.61 (3.11-4.11) | 0.11 |
|  | **OVERALL MEAN** | **3.30 (2.80-3.80)** | **3.46 (3.02-3.91)** | **0.21** |
|  |  |  |  |  |
| Sexual misconduct | Number of physicians | 219 | 532 |  |
|  | Staff | 3.79 (3.32-4.26) | 3.59 (3.13-4.06) | 0.03 |
|  | Punctuality | 3.26 (2.80-3.72) | 3.06 (2.61-3.50) | 0.03 |
|  | Helpfulness | 3.63 (3.10-4.15) | 3.29 (2.78-3.81) | <0.001 |
|  | Knowledge | 3.80 (3.29-4.32) | 3.48 (2.97-3.99) | <0.001 |
|  | **OVERALL MEAN** | **3.63 (3.17-4.08)** | **3.35 (2.91-3.80)** | **0.003** |
|  |  |  |  |  |
| Mental illness | Number of physicians | 4 | 747 |  |
|  | Staff | 4.03 (2.72-4.35) | 3.66 (3.20-4.12) | 0.58 |
|  | Punctuality | 3.26 (1.98-4.55) | 3.13 (2.68-3.57) | 0.83 |
|  | Helpfulness | 4.14 (2.69-5.6) | 3.39 (2.88-3.91) | 0.30 |
|  | Knowledge | 4.38 (2.95-5.81) | 3.58 (3.07-4.08) | 0.26 |
|  | **OVERALL MEAN** | **3.97 (2.70-5.25)** | **3.44 (2.99-3.88)** | 0.40 |
|  |  |  |  |  |
| Drugs and Alcohol | Number of physicians | 21 | 730 |  |
|  | Staff | 3.97 (3.27-4.68) | 3.65 (3.19-4.11) | 0.28 |
|  | Punctuality | 3.49 (2.80-4.18) | 3.12 (2.67-3.56) | 0.18 |
|  | Helpfulness | 3.74 (2.95-4.53) | 3.40 (2.89-3.92) | 0.30 |
|  | Knowledge | 3.91 (3.14-4.68) | 3.58 (3.08-4.09) | 0.30 |
|  | **OVERALL MEAN** | **3.79 (3.10-4.47)** | **3.44 (3.00-3.89)** | **0.22** |
|  |  |  |  |  |
| Fraudulent behavior | Number of physicians | 115 | 636 |  |
|  | Staff | 3.39 (2.87-3.90) | 3.65 (3.20-4.11) | 0.03 |
|  | Punctuality | 3.12 (2.68-3.57) | 3.12 (2.68-3.57 | 0.03 |
|  | Helpfulness | 3.10 (2.52-3.67) | 3.40 (2.89-3.91) | 0.02 |
|  | Knowledge | 3.26 (2.70-3.83) | 3.58 (3.08-4.09) | 0.02 |
|  | **OVERALL MEAN** | **3.15 (2.65-3.65)** | **3.44 (2.30-3.89)** | **0.01** |
|  |  |  |  |  |
| Conviction of a crime | Number of physicians | 43 | 707 |  |
|  | Staff | 3.37 (2.77-4.00) | 3.66 (3.20-4.12) | 0.15 |
|  | Punctuality | 3.06 (2.48-3.65) | 3.13 (2.69-3.58) | 0.73 |
|  | Helpfulness | 3.32 (2.65-4.00) | 3.41 (2.90-3.92) | 0.71 |
|  | Knowledge | 3.54 (2.88-4.20) | 3.60 (3.09-4.10) | 0.85 |
|  | **OVERALL MEAN** | **3.33 (2.75-3.92)** | **3.45 (3.00-3.90)** | **0.54** |
|  |  |  |  |  |
| Unprofessional behavior | Number of physicians | 173 | 578 |  |
|  | Staff | 3.68 (3.20-415) | 3.66 (3.19-4.12) | 0.81 |
|  | Punctuality | 3.08 (2.61-3.54) | 3.16 (2.71-3.61) | 0.39 |
|  | Helpfulness | 3.23 (2.70-3.76) | 3.50 (2.98-4.02) | 0.02 |
|  | Knowledge | 3.42 (2.90-3.94) | 3.68 (3.17-4.19) | 0.020 |
|  | **OVERALL MEAN** | **3.35 (2.89-3.81)** | **3.35 (2.89-3.81)** | **0.10** |
|  |  |  |  |  |
| Miscellaneous/Unclear/Other | Number of physicians | 168 | 583 |  |
|  | Staff | 3.52 (3.05-4.00) | 3.72 (3.26-4.18) | 0.05 |
|  | Punctuality | 2.94 (2.48-3.40) | 3.21 (2.76-3.65) | 0.01 |
|  | Helpfulness | 3.28 (2.75-3.82) | 3.46 (2.94-3.98) | 0.11 |
|  | Knowledge | 3.48 (2.96-4.00) | 3.634 (3.13-4.14) | 0.16 |
|  | **OVERALL MEAN** | **3.31 (2.85-3.77)** | **3.51 (3.06-3.96)** | **0.04** |
|  |  |  |  |  |
| Revocation | Number of physicians | 112 | 638 |  |
|  | Staff | 3.59 (3.03-4.14) | 3.66 (3.21-4.12) | 0.61 |
|  | Punctuality | 2.98 (2.45-3.51) | 3.13 (2.69-3.57) | 0.32 |
|  | Helpfulness | 3.44 (2.83-4.05) | 3.14 (2.90-3.93) | 0.86 |
|  | Knowledge | 3.56 (2.96-4.16) | 3.60 (3.09-4.10) | 0.83 |
|  | **OVERALL MEAN** | **3.37 (2.84-3.91)** | **3.45 (3.01-3.90)** | **0.59** |
|  |  |  |  |  |
| Surrender | Number of physicians | 44 | 704 |  |
|  | Staff | 3.13 (2.68-3.57) | 3.66 (3.20-4.12) | 0.59 |
|  | Punctuality | 3.31 (2.69-3.93) | 3.13 (2.68-3.57) | 0.43 |
|  | Helpfulness | 3.53 (2.82-4.24) | 3.41 (2.89-3.92) | 0.63 |
|  | Knowledge | 3.67 (2.97-4.37) | 3.59 (3.09-4.10) | 0.75 |
|  | **OVERALL MEAN** | **3.61 (2.99-4.23)** | **3.45 (3.00-3.89)** | **0.48** |
|  |  |  |  |  |
| Suspension | Number of physicians | 387 | 364 |  |
|  | Staff | 3.73 (3.23-4.19) | 3.56 (3.09-4.03) | 0.07 |
|  | Punctuality | 3.19 (2.74-3.63) | 3.05 (2.59-3.50) | 0.13 |
|  | Helpfulness | 3.52 (3.00-4.03) | 3.28 (2.76-3.80) | 0.02 |
|  | Knowledge | 4.33 (3.61-5.06) | 3.46 (2.94-3.97) | 0.01 |
|  | **OVERALL MEAN** | **3.54 (3.10-3.99)** | **3.33 (2.88-3.79)** | **0.02** |
|  |  |  |  |  |
| Restriction | Number of physicians | 240 | 511 |  |
|  | Staff | 3.69 (3.23-4.14) | 3.60 (3.12-4.08) | 0.37 |
|  | Punctuality | 3.44 (2.92-3.95) | 3.03 (2.57-3.49) | 0.15 |
|  | Helpfulness | 3.44 (2.92-3.95) | 3.44 (2.92-3.95) | 0.30 |
|  | Knowledge | 3.60 (3.10-4.11) | 3.56 (3.03-4.08) | 0.62 |
|  | **OVERALL MEAN** | **3.48 (3.03-3.93)** | **3.37 (2.90-3.84)** | **0.25** |
|  |  |  |  |  |
| Retraining | Number of physicians | 231 | 520 |  |
|  | Staff | 3.66 (3.21-4.12) | 3.67 (3.18-4.15) | 0.99 |
|  | Punctuality | 3.12 (2.68-3.57) | 3.15 (2.69-3.62) | 0.77 |
|  | Helpfulness | 3.42 (2.90-3.93) | 3.40 (2.86-3.93) | 0.84 |
|  | Knowledge | 3.60 (3.07-4.12) | 3.60 (3.07-4.12) | 0.99 |
|  | **OVERALL MEAN** | **3.45 (2.98-3.92)** | **3.45 (2.98-3.92)** | **0.99** |
|  |  |  |  |  |
| Counselling | Number of physicians | 80 | 669 |  |
|  | Staff | 3.69 (3.19-4.19) | 3.66 (3.20-4.12) | 0.82 |
|  | Punctuality | 3.07 (2.59-3.56) | 3.15 (2.70-3.60) | 0.54 |
|  | Helpfulness | 3.32 (2.70-3.60) | 3.44 (2.92-3.95) | 0.47 |
|  | Knowledge | 3.47 (2.92-4.02) | 3.63 (3.12-4.13) | 0.32 |
|  | **OVERALL MEAN** | **3.40 (2.91-3.88)** | **3.47 (3,02-3.92)** | **0.57** |
|  |  |  |  |  |
| Formal Reprimand | Number of physicians | 375 | 375 |  |
|  | Staff | 3.51 (3.03-4.00) | 3.68 (3.22-4.14) | 0.08 |
|  | Punctuality | 3.00 (2.52-3.47) | 3.15 (2.71-3.59) | 0.11 |
|  | Helpfulness | 3.23 (2.68-3.77) | 3.43 (2.91-2.95) | 0.06 |
|  | Knowledge | 3.54 (2.97-4.10) | 3.61 (3.11-4.12) | 0.11 |
|  | **OVERALL MEAN** | **3.42 (2.92-3.92)** | **3.47 (3.03-3.92)** | **0.06** |
|  |  |  |  |  |
| Fine | Number of physicians | 72 | 185 |  |
|  | Staff | 3.65 (3.20-4.11) | 3.74 (3.24-4.23) | 0.45 |
|  | Punctuality | 3.12 (2.67-3.56) | 3.24 (2.76-3.72) | 0.26 |
|  | Helpfulness | 3.40 (2.89-3.92) | 3.45 (2.89-4.00) | 0.74 |
|  | Knowledge | 3.58 (3.08-4.08) | 3.65 (3.11-4.19) | 0.61 |
|  | **OVERALL MEAN** | **3.44 (3.00-3.89)** | **3.53 (3.05-4.01)** | **0.42** |
|  |  |  |  |  |
| Other Punishment | Number of physicians | 72 | 676 |  |
|  | Staff | 3.65 (3.13-4.16) | 3.67 (3.21-4.13) | 0.86 |
|  | Punctuality | 3.16 (2.66-3.66) | 3.13 (2.68-3.57) | 0.80 |
|  | Helpfulness | 3.32 (2.74-3.90) | 3.42 (2.91-3.94) | 0.52 |
|  | Knowledge | 3.54(2.97-4.11) | 3.60 (3.10-4.11) | 0.70 |
|  | **OVERALL MEAN** | **3.42 (2.92-3.92)** | **3.46 (3.01-3.90)** | **0.79** |
