# Supplementary material for: Association Between Web-Based Physician Ratings and Physician Disciplinary Convictions: Retrospective Observational Study
Source: J Med Internet Res. 2020 May 14;22(5):e16708. doi: 10.2196/16708 (PMC7256745; doi:10.2196/16708)
Supplement: Multimedia Appendix 2 [file jmir_v22i5e16708_app2.docx]

|  | **Original analysis: physicians with at least 1 rating** | **Sensitivity analysis: physicians with at least 5 ratings** | **Sensitivity analysis: physicians with at least 10 ratings** |
| --- | --- | --- | --- |
| **ALL MISCONDUCT** | | | |
| Number of case control pairs | 312 | 227 | 168 |
| Disciplined cases, mean rating (95% CI) | 3.52 (3.53-3.71) | 3.61 (3.51-3.71) | 3.52 (3.40-3.62) |
| Undisciplined controls, mean rating (95% CI) | 4.0 (3.91-4.08) | 4.01 (3.92-4.09) | 4.01 (3.91-4.10) |
| *P*-value | P<0.001 | P<0.001 | P<0.001 |
|  |  |  |  |
| **STANDARD OF CARE BREACH** | | | |
| Number of case control pairs | 113 | 82 | 62 |
| Disciplined cases, mean rating (95% CI) | 3.57 (3.42-3.72) | 3.54 (3.38-3.76) | 3.45 (3.26-3.63) |
| Undisciplined controls, mean rating (95% CI) | 3.98 (3.84-4.12) | 4.04 (2.91-4.17) | 4.02 (3.87-4.17) |
| *P*-value | P<0.001 | P<0.001 | P<0.001 |
|  |  |  |  |
| **INAPPROPRIATE PRESCRIBING** | | | |
| Number of case control pairs | 55 | 43 | 32 |
| Disciplined cases, mean rating (95% CI) | 3.50 (3.29-3.71) | 3.46 (3.22-3.69) | 3.38 (3.09-3.67) |
| Undisciplined controls, mean rating (95% CI) | 3.97 (3.79-4.16) | 3.94 (3.75-4.13) | 3.91 (3.70-4.12) |
| *P*-value | P=0.001 | P<0.001 | P<0.001 |
|  |  |  |  |
| **SEXUAL MISCONDUCT** | | | |
| Number of case control pairs | 90 | 59 | 42 |
| Disciplined cases, mean rating (95% CI) | 3.83 (3.67-3.99) | 3.89 (3.74-4.05) | 3.79 (3.61-3.97) |
| Undisciplined controls, mean rating (95% CI) | 3.86 (3.68-4.04) | 3.94 (3.76-4.11) | 3.92 (3.70-4.13) |
| *P*-value | P=0.81 | P=0.69 | P=0.36 |
|  |  |  |  |
| **UNPROFESSIONAL BEHAVIOUR** | | | |
| Number of case control pairs | 62 | 43 | 30 |
| Disciplined cases, mean rating (95% CI) | 3.43 (3.22-3.64) | 3.61 (3.39-3.83) | 3.50 (3.23-3.77) |
| Undisciplined controls, mean rating (95% CI) | 4.03 (3.86-4.21) | 3.98 (3.77-4.19) | 4.02 (3.8-4.24) |
| *P*-value | P<0.001 | P=0.02 | P=0.001 |
|  |  |  |  |
| **LICENSE SUSPENSION** | | | |
| Number of case control pairs | 191 | 136 | 105 |
| Disciplined cases, mean rating (95% CI) | 3.71 (3.60-3.83) | 3.72 (3.60-3.85) | 3.66 (3.51-3.80) |
| Undisciplined controls, mean rating (95% CI) | 3.99 (3.88-4.10) | 4.01 (3.91-4.12) | 4.01 (3.90-4.13) |
| *P-*value | P<0.001 | P<0.001 | P<0.001 |
|  |  |  |  |
| **RESTRICTION** | | | |
| Number of case control pairs | 115 | 86 | 67 |
| Disciplined cases, mean rating (95% CI) | 3.69 (3.55-3.83) | 3.73 (3.60-3.87) | 3.68 (3.53-3.83) |
| Undisciplined controls, mean rating (95% CI) | 3.91 (3.78-4.05) | 3.92 (3.78-4.06) | 3.96 (3.82-4.10) |
| *P*-value | P=0.03 | P=0.08 | P=0.01 |
|  |  |  |  |
| **MANDATORY RETRAINING/EDUCATION** | | | |
| Number of case control pairs | 120 | 90 | 70 |
| Disciplined cases, mean rating (95% CI) | 3.61 (3.48-3.73) | 3.59 (3.46-3.71) | 3.48 (3.34-3.63) |
| Undisciplined controls, mean rating (95% CI) | 3.98 (3.85-4.12) | 3.91 (3.77-4.06) | 3.95 (3.80-4.10) |
| *P*-value | P<0.001 | P=0.001 | P<0.001 |
|  |  |  |  |
| **FORMAL REPRIMAND** | | | |
| Number of case control pairs | 144 | 99 | 78 |
| Disciplined cases, mean rating (95% CI) | 3.58 (3.45-3.71) | 3.59 (3.45-3.74) | 3.55 (3.38-3.72) |
| Undisciplined controls, mean rating (95% CI) | 4.02 (3.89-4.14) | 4.03 (3.90-4.16) | 4.02 (3.89-4.16) |
| *P*-value | P<0.001 | P<0.001 | P<0.001 |
|  |  |  |  |
| **FINE** | | | |
| Number of case control pairs | 254 | 187 | 141 |
| Disciplined cases, mean rating (95% CI) | 3.60 (3.50-3.70) | 3.59 (3.48-3.70) | 3.50 (3.38-3.63) |
| Undisciplined controls, mean rating (95% CI) | 4.02 (3.93-4.11) | 4.02 (3.92-4.12) | 4.03 (3.92-4.14) |
| *P*-value | P<0.001 | P<0.001 | P<0.001 |
|  |  |  |  |
