# Supplementary material for: Association Between Web-Based Physician Ratings and Physician Disciplinary Convictions: Retrospective Observational Study
Source: J Med Internet Res. 2020 May 14;22(5):e16708. doi: 10.2196/16708 (PMC7256745; doi:10.2196/16708)
Supplement: Multimedia Appendix 3 [file jmir_v22i5e16708_app3.docx]

|  | **Original analysis: physicians with at least 1 rating** | **Physicians with at least 5 ratings** | **Physicians with at least 10 ratings** |
| --- | --- | --- | --- |
| **STANDARD OF CARE** |  | | |
| **Disciplined physicians, specific misconduct, number** | 243 | 137 | 118 |
| **Disciplined physicians, specific misconduct, mean rating (95% CI)** | 3.46 (3.00-3.92) | 3.47 (3.05-4.02) | 3.45 (2.99-3.91) |
| **All other disciplined physicians, mean rating (95% CI)** | 3.45 (3.00-3.90) | 3.45 (3.04-3.87) | 3.43 (2.98-3.88) |
| **All other disciplined physicians, number** | 508 | 253 | 213 |
| ***P*-value** | P=0.92 | P=0.80 | P=0.85 |
|  |  |  |  |
| **INAPPROPRIATE PRESCRIBING** |  | | |
| **Disciplined physicians, specific misconduct, number** | 114 | 71 | 60 |
| **Discipline physicians, specific misconduct, mean rating (95% CI)** | 3.45 (2.96-3.94) | 3.47 (3.01-3.92) | 3.44 (2.95-3.93) |
| **All other disciplined physicians, mean rating (95% CI)** | 3.45 (3.01-3.90) | 3.46 (3.05-3.87) | 3.44 (2.99-3.88) |
| **All other disciplined physicians, number** | 637 | 319 | 271 |
| ***P*-value** | P=0.98 | P=0.93 | P=0.61 |
|  |  |  |  |
| **SEXUAL MISCONDUCT** |  | | |
| **Disciplined physicians, specific misconduct, number** | 219 | 104 | 91 |
| **Disciplined physicians, specific misconduct, mean rating (95% CI)** | 3.63 (3.17-4.08) | 3.70 (3.28-4.12) | 3.69 (3.23-4.15) |
| **All other disciplined physicians, mean rating (95% CI)** | 3.35 (2.91-3.80) | 3.37 (2.96-3.77) | 3.37 (2.93-3.81) |
| **All other disciplined physicians, number** | 532 | 286 | 240 |
| ***P*-value** | P=0.003 | P<0.001 | P<0.001 |
|  |  |  |  |
| **FRAUDULENT BEHAVIOUR** |  | | |
| **Disciplined physicians, specific misconduct, number** | 115 | 59 | 50 |
| **Disciplined physicians, specific misconduct, mean rating (95% CI)** | 3.15 (2.65-3.65) | 3.30 (2.83-3.76) | 3.25 (2.73-3.76) |
| **All other disciplined physicians, mean rating (95% CI)** | 3.44 (2.30-3.89) | 3.46 (3.05-3.87) | 3.41 (2.96-3.86) |
| **All other disciplined physicians, number** | 636 | 331 | 281 |
| ***P*-value** | P=0.01 | P=0.15 | P=0.17 |
|  |  | | |
| **SUSPENSION** |  | | |
| **Disciplined physicians, specific misconduct, number** | 387 | 152 | 125 |
| **Disciplined physicians, specific misconduct, mean rating (95% CI)** | 3.54 (3.10-3.99) | 3.53 (3.12-3.94) | 3.50 (3.06-3.95) |
| **All other disciplined physicians, mean rating (95% CI)** | 3.33 (2.88-3.79) | 3.3 (2.90-3.74) | 3.32 (2.86-3.77) |
| **All other disciplined physicians, number** | 364 | 238 | 206 |
| ***P*-value** | P=0.02 | P=0.02 | P=0.06 |
